# Initial Development of Automated Machine Learning-Assisted Prediction Tools for Aryl Hydrocarbon Receptor Activators

**DOI:** 10.3390/pharmaceutics16111456

**Published:** 2024-11-15

**Authors:** Paulina Anna Wojtyło, Natalia Łapińska, Lucia Bellagamba, Emidio Camaioni, Aleksander Mendyk, Stefano Giovagnoli

**Affiliations:** 1 Department of Pharmaceutical Sciences, University of Perugia, via del Liceo 1, 06123 Perugia, Italy; lucia.bellagamba@studenti.unipg.it (L.B.); emidio.camaioni@unipg.it (E.C.); stefano.giovagnoli@unipg.it (S.G.); 2 Department of Pharmaceutical Technology and Biopharmaceutics, Jagiellonian University Medical College, 30-688 Kraków, Poland; natalia.czub@uj.edu.pl (N.Ł.); aleksander.mendyk@uj.edu.pl (A.M.)

**Keywords:** aryl hydrocarbon receptor, machine learning, AutoML, QSAR model

## Abstract

**Background**: The aryl hydrocarbon receptor (AhR) plays a crucial role in immune and metabolic processes. The large molecular diversity of ligands capable of activating AhR makes it impossible to determine the structural features useful for the design of new potent modulators. Thus, in the field of drug discovery, the intricate nature of AhR activation necessitates the development of novel tools to address related challenges. **Methods**: In this study, quantitative structure–activity relationship (QSAR) models of classification and regression were developed with the objective of identifying the most effective method for predicting AhR activity. The initial dataset was obtained by combining the ChEMBL and WIPO databases which contained 978 molecules with EC_50_ values. The predictive models were developed using the automated machine learning platform mljar according to a 10-fold cross validation (10-CV) testing procedure. **Results**: The classification model demonstrated an accuracy value of 0.760 and F1 value of 0.789 for the test set. The root-mean-squared error (RMSE) was 5444, and the coefficient of determination (R^2^) was 0.208 for the regression model. The Shapley Additive Explanations (SHAP) method was then employed for a deeper comprehension of the impact of the variables on the model’s predictions. As a practical application for scientific purposes, the best performing classification model was then used to develop an AhR web application. This application is accessible online and has been implemented in Streamlit. **Conclusions**: The findings may serve as a foundation in prompting further research into the development of a QSAR model, which could enhance comprehension of the influence of ligand structure on the modulation of AhR activity.

## 1. Introduction

The aryl hydrocarbon receptor (AhR) is a member of the family of basic helix–loop–helix (bHLH) ligand-dependent transcription factors, which plays a significant role in regulating gene expression, which affects several biological processes, including inflammation and homeostasis [1]. The AhR is mainly found in cytoplasm as an inactive complex with chaperone proteins, such as HSP90 and XAP2. The AhR is freed from the complex as a result of ligand binding and migrates into the nucleus where it binds to the AhR nuclear transport (ARNT) protein, thereby promoting the modulation of cellular homeostasis and the immune system [2]. AhR modulation also regulates cell cycle, cell differentiation and chemical and microbial defense. Since its discovery, the AhR has been identified as a sensor for xenobiotics, capable of inducing toxic effects upon activation [3]. The most extensively studied exogenous ligands for the AhR are halogenated aromatic hydrocarbons (HAHs), particularly 2,3,7,8-tetrachlorodibenzo-p-dioxin (TCDD), the high-affinity ligand for the AhR. However, it also binds a wide array of substances, including naturally occurring flavonoids, polyphenols and indoles, which are byproducts of microbial metabolism [4]. This molecular diversity of AhR modulators further complicates the complex path towards the design of novel receptor agonists. At the same time, such AhR promiscuity makes it a promising therapeutic target for a wide range of purposes. This explains the extensive research digging into the role of the AhR in numerous metabolic and pathological processes, including cancer, cystic fibrosis, epithelial barrier function, autoimmune disorders such as psoriasis and more recently, its influence on the brain–gut axis in Alzheimer’s disease and Parkinson’s disease [5,6,7,8].

Examples of AhR ligand promiscuity are Tapinarof (3,5-dihydroxy-4-isopropylstilbene), a nonsteroidal drug, approved by the US Food and Drug Administration in 2022 for the treatment of psoriasis [9] and indole-3-carboxaldehyde (3-IALD), an endogenous tryptophan microbial metabolite that received the orphan drug designation in 2023 by the European Medicines Agency for the treatment of primary Cytotoxic T-Lymphocyte Antigen 4 (CTLA-4) checkpoint-related immunodeficiencies [10]. Both compounds are AhR agonists and exhibit anti-inflammatory activity in spite of their structural diversity [11,12].

As a consequence of the above-mentioned promiscuity as well as the complex AhR processes, a lack of clear correlation between AhR modulation and biological effects is often observed. In fact, AhR modulation can result in not always predictable and sometimes contrasting activities related to the ligand molecular scaffold and chemical nature [13].

It is clear that structure-based drug discovery methods greatly fail in AhR ligand design and modeling. Although highly efficient high-throughput screening (HTS) techniques are today available in drug discovery, their value comes to an end when confronting highly promiscuous targets, such as the AhR. Therefore, increasing efforts are focusing on the development of innovative methods based on artificial intelligence (AI) tools. This relatively new frontier of research is expanding to areas such as pharmaceutical manufacturing and drug discovery [14].

In particular, machine learning (ML) is a powerful technique based on automatic learning processes that can be applied to large datasets without the necessity for extensive computational resources [15]. Two different ML approaches can be employed, referred to as unsupervised or supervised ML. The former involves ML algorithms looking for patterns and structures in input data without explicit supervision or labeled output; on the contrary, in supervised ML, algorithms are trained to associate input data with specific output labels. In supervised learning, the system parameters are modified in accordance with the discrepancy between the predicted output and the desired output [16]. Compared to unsupervised ML, supervised ML requires large amounts of labeled or annotated data to develop its predictive capability. Moreover, within the ML approach, two distinct methods may be selected: classification and regression. Classification is the task of automatically assigning labels to unlabeled examples using a classification algorithm. This algorithm learns from labeled examples to create a model that can output labels directly or provide scores for label deduction [17]. Classification can be binary (two classes) or multiclass (three or more classes). In contrast, regression involves predicting continuous values from unlabeled examples. A regression algorithm learns from labeled data to produce a model that can output a real-valued label for new unlabeled inputs [18]. Several drugs have been discovered with the assistance of AI and have advanced significantly in clinical trials. Some of them, like Baricitinib and Halicin, have achieved FDA and EMA approval [18].

Few studies have been undertaken to predict AhR activity combining QSAR and ML. Zhu et al. presented a virtual screening protocol that integrated ligand-based and structure-based screening with supervised ML to identify agonistic effects on AhR activity and screened approximately 8000 structures from a pesticide database. The results demonstrated the activation of the AhR by 16 compounds, a finding that was subsequently validated in a zebrafish model [19]. Goya-Jorge et al. developed a series of classification models utilizing a comprehensive TOX21 database provided by the National Toxicology Program [20]. In vitro experimental validation substantiated the predictive capacity of these models, identifying benzothiazoles as the most prominent group among AhR agonists. Matsuzaka’s work proposed DeepSnap-DL, a deep learning (DL)-aided QSAR analysis tool, to construct prediction models of AhR activation on an in-house database containing 201 chemicals. Compared with conventional ML, DeepSnap–DL demonstrated high-performance prediction of AhR-induced hepatotoxicity [21]. Although these approaches performed well on molecular congeners, their versatility has yet to be demonstrated. It should also be noted that none of these tools are available as free online sources.

In this work, an initial study was undertaken to investigate supervised ML approaches to obtain a predictive model of AhR activation with acceptable performance. A number of different models were built by automated machine learning (AutoML) methods to discern between regression or classification approaches [18]. AutoML is an automation process designed to streamline the tasks involved in the creation of ML models to facilitate and accelerate model development process. It encompasses data preparation, model selection and its associated hyperparameters, evaluation, and implementation. The optimized model was then implemented in the form of a web-based application for wider use.

## 2. Materials and Methods

### 2.1. Database

The working database was constructed using data from the following sources: ChEMBL [22], PubChem [23], the scientific literature [12,24,25,26,27,28,29,30,31,32,33] and patents in the World Intellectual Property Organization (WIPO) database [34], with the last accession in July 2024. The information required for the construction of the database included the molecular name or structure, which enabled the generation of the molecular representation SMILES (Simplified Molecular Input Line Entry System). The necessary biological data comprised EC_50_ values and the assay protocol, which provided crucial information such as the utilized cell line and the method employed to obtain the biological data, resulting in a final number of 978 molecules. The molecules included in the database were tested on the following human cell lines: Human Hepatocellular Carcinoma Cell Line (HepG2), Human Embryonic Kidney 293 Cell Line (HEK293), Human Histiocytic Lymphoma Cell Line U937 (U937), Human Hepatoma Cell Line 7 (Huh-7), Human Colorectal Adenocarcinoma Cell Line 29 (HT29) and Michigan Cancer Foundation-7 (MCF7). The luciferase Assay, the Ethoxyresorufin-O-deethylase Assay and the Nuclear Translocation Assay were employed to calculate the EC_50_ values.

The data underwent processing with the aid of a variety of offline tools, namely DataWarrior, version 6.2.5 [35], the Pandas library of the Python programming language, version 3.12.4 [36] and the RDKit tool, version 2024.03 [37].

The preprocessing procedure entailed the removal of errors, including those in the form of duplicate and/or missing values. Additionally, curation of the database involved the conversion of non-numerical values into numbers, specifically those representing biological assays and cell lines. In the present study, only two-dimensional molecular descriptors were calculated with the Mordred package, version 1.2.0 [38].

### 2.2. QSAR Model

To ascertain which model would perform more effectively with the obtained database, classification and regression models were developed in parallel and evaluated. The modeling was performed with the mljar-supervised tool, which is within the domain of AutoML [39]. In order to evaluate the efficacy of classification and regression methods, a series of models were constructed using a 10-fold cross-validation scheme (10-CV) on the training set of selected datasets.

In the classification method, the estimated threshold for the EC_50_ value was 1000 nM. The database was divided into a training set and a test set using a random split of 80/20. A regression model was constructed using the database, with the training set containing 701 molecules and the test set having 166 molecules, excluding 111 other molecules. The training set was used to develop the model according to 10-fold cross-validation, and the test set was used for external evaluation of the model.

The evaluation of the classification model’s performance was conducted employing the following main metrics: accuracy, precision, recall, F1 score and Matthews correlation coefficient (MCC). For reference, see Equations (1)–(5).

(1) $$accuracy=\frac{TP+TN}{TP+TN+FP+FN}$$

(2) $$precision=\frac{TP}{TP+FP}$$

(3) $$recall=\frac{TP}{TP+FN}$$

(4) $$MCC=\frac{TP\times TN-FP\times FN}{\sqrt{\left(TP+FP\right)\left(TP+FN\right)\left(TN+FP\right)\left(TN+FN\right)}}$$

where *TP* = true positive, *TN* = true negative, *FP* = false positive and *FN* = false negative.

(5) $$F1=\frac{2\times precision\times recall}{precision+recall}$$

With the aim of assessing the performance of the regression model, three goodness-of-fit metrics were considered: root-mean-square error (*RMSE*), normalized root-mean-square error (*NRMSE*) and coefficient of determination (*R*^2^). They are presented below in Equations (6)–(8).

(6) $$RMSE=\sqrt{\frac{\sum_{i=1}^{n}{\left(pred_{i}-obs_{i}\right)}^{2}}{n}}$$

where *pred_i_* and *obs_i_* are predicted and observed values, respectively, *i* is the data record number, and n is the total number of records.

(7) $$NRMSE=\frac{RMSE}{obs_{max}-obs_{min}}\times 100\%$$

where *RMSE* is the root-mean-square-error calculated for the model, and *obs_max_* and *obs_min_* are the observed maximal and minimal values results.

(8) $$R^{2}=1-\frac{SS_{res}}{SS_{tot}}=1-\frac{\sum_{i=1}^{n}{\left(pred_{i}-obs\right)}^{2}}{\sum_{i=1}^{n}{\left(obs_{i}-obs\right)}^{2}}$$

Here, *R*^2^ is the coefficient of determination, *SS_res_* is the sum of squares of the residual errors, *SS_tot_* is the total sum of the errors, *obs_i_* and *pred_i_* are the observed and predicted values, and *obs* is the arithmetical mean of the observed values. The best model is one that presents higher *R*^2^ values.

### 2.3. SHAP Analysis

Explainable Artificial Intelligence (XAI) is a concept that has grown with the rapid development of artificial intelligence in recent years. By assumption, a model created by large computational resources creates models that are difficult to interpret. XAI provides insight into how the model works. As part of this study, we used SHAP analysis. SHAP (Shapley Additive Explanations), introduced by Lloyd Shapley in 1952 [40], is based on game theory, verifying the distribution of rewards in a game between cooperating players. This distribution should be proportional to their contribution to the outcome, while satisfying the criteria of efficiency, additivity, symmetry and detection of the presence of a zero player. A unique solution to this problem is the Shapley value, which distributes the reward based on each player’s marginal contribution, ensuring fairness [41].

The Shapley value *ϕ_i_* for the feature *i* is calculated as follows:

$$\phi_{i}=\sum_{S\subseteq N\backslash \backslash \{i\}}\frac{\left|S\right|!\left(\left|N\right|-\left|S\right|-1\right)!}{\left|N\right|!}\left[f(S\cup \left\{i\right\}-f\left(S\right)\right]$$

where *N* is the set of all features, *S* represents each possible subset of *N* that does not include *i*, *f(S)* is the model prediction using only the features in *S*, *f(S∪{i})* is the prediction with feature *i* added and *|S|!* and *(|N| − |S| − 1)!* are factorial terms that weight each subset’s contribution.

In Shapley value analysis, the efficiencies in the total contributions from all features should sum to the model’s prediction. This ensures that all feature contributions are fully accounted for, with no surplus or deficit.

$$\sum_{i\in N}\varphi_{i}\left(\nu \right)=\nu \left(N\right)$$

Symmetry refers to the concept according to which when two features contribute equally, they should have equal Shapley values, ensuring the fair representation of redundant or similar features.

$$val\left(S\cup \left\{j\right\}\right)=val(S\cup \left\{k\right\})$$

The concept of additivity ensures that if a subset’s features or models are considered, the Shapley values are the sum of each individual feature or model, promoting the consistency of different models or feature sets.

$$\phi_{j}+\phi_{j}^{+}$$

The null player (“zero player”) is a feature that has no impact on any prediction. Regardless of its presence or absence, the predicted outcome remains unchanged.

$$v\left(S\cup \left\{i\right\}\right)=(S)$$

In the case of the QSAR model, SHAP analysis was used to determine the impact of the most relevant descriptors in order to easily determine the applicability domain (AD) in which the model leads to reliable predictions. The analysis was performed in the Python environment, using a framework developed elsewhere [42] that was further extended with a wrapper from the mljar package.

The model was explained using the kernel-based SHAP explainer, which is a model-agnostic technique and can be applied to any machine learning model, especially complex ensemble models. The bees swarm plot was drawn as a result of using the summary_plot() function.

## 3. Results

### 3.1. Database

**Figure 1:**
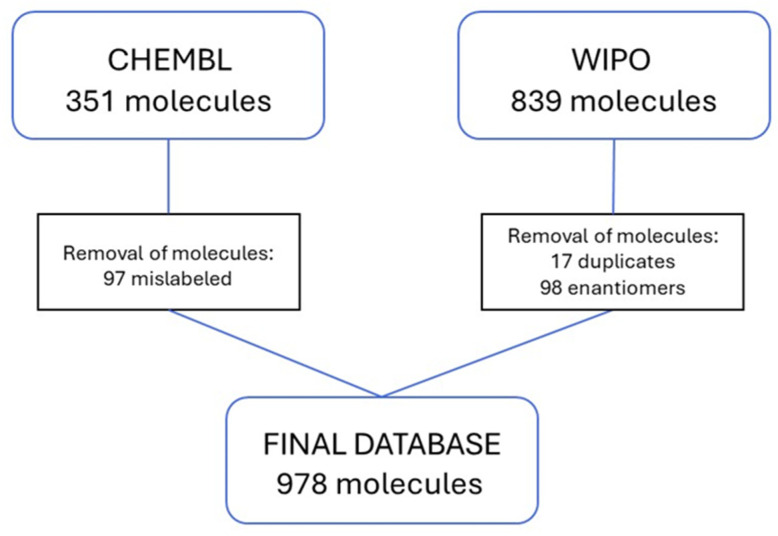
Scheme of dataset composition.

In order to obtain the largest number of molecules described by the EC_50_ value, an exhaustive search followed by comprehensive analysis was conducted on a range of available sources. The ChEMBL database was searched for the target ID: The CHEMBL3201 entry, designated “Aryl hydrocarbon receptor” and corresponding to the organism “Homo sapiens”, was downloaded and subjected to a verification process involving the examination of cited sources to ascertain the veracity of the results and to identify and eliminate potential errors. The database for the described target included 351 records; however, following a visual and detailed examination of the scientific literature and the PubChem resource referenced in CHEMBL, only 254 molecules met the requisite criteria. Molecules that were removed from the database were excluded due to errors, such as duplication or incorrect organism marking. The decision was made to utilize only 2D-descriptors, and therefore any chiral molecules that appeared as enantiomers were subsequently eliminated from consideration. An analysis of sources cited by CHEMBL revealed the presence of additional structures in published patents, which were incorporated into the database. A search of the WIPO database was conducted for the term “Aryl hydrocarbon receptor”. The patents obtained through this system were subjected to a review process. In this step, the molecules that fulfilled the criteria for inclusion in the database, as previously defined for the ChEMBL database, were selected. The SMILES and EC_50_ data from the patents were extracted manually. A search conducted through the WIPO database enabled the inclusion of further 839 molecules, and after removing redundant molecules and enantiomeric pairs, the final database comprised 978 structures (Figure 1). The database utilized in this study is accessible in the Supplementary Materials.

The EC_50_ values of the molecules included in the database resulted from three common biological assays. Each of these assays provides complementary information about AhR activation. The Luciferase Assay offers a high-throughput option for screening compounds (Luminescence Assay), the Ethoxyresorufin-O-deethylase Assay (Fluorescence EROD Assay) directly measures enzyme activity induced by AhR and the Nuclear Translocation Assay (Fluorescence NT Assay) provides visual confirmation of receptor activation. Together, they offer a comprehensive approach to studying AhR function and screening potential drugs or environmental toxicants to be included in this study. The number of compounds labeled using the luciferase, EROD and NT techniques was 802, 28 and 148, respectively. Another variable incorporated in the database is the diverse range of cell lines used in the above-mentioned assays. All molecules included in the database were tested on human cell lines. The largest number of molecules were tested on the Human Hepatocellular Carcinoma Cell Line (HepG2) with 648 molecules, followed by Human Embryonic Kidney 293 Cell Line (HEK293) with 148 molecules, Human Histiocytic Lymphoma Cell Line U937 (U937) with 98 molecules, Human Hepatoma Cell Line 7 (Huh-7) with 51 molecules, Human Colorectal Adenocarcinoma Cell Line 29 (HT29) with 24 molecules and Michigan Cancer Foundation-7 (MCF7) with 9 molecules (Table 1).

**Figure 2:**
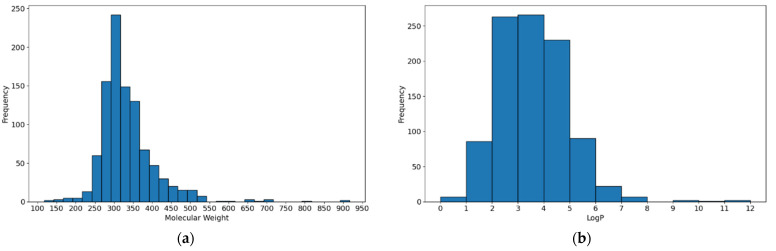
Frequency of molecular weight (**a**) and logP (**b**) values in curated database.

One challenge in the construction of the database was handling those molecules for which the EC_50_ value was expressed as a numerical threshold or range (Table 2). This issue was solved on an individual basis for each model, as described in Section 3.1.1 and Section 3.1.2. Moreover, in order to provide an in-depth comprehension of the molecules within the database, Figure 2 illustrates the distribution of molecular weight and logP values within the database. Both logP and molecular weight display unimodal, right-skewed distributions. This demonstrates the substantial complexity of the analyzed problem, since a non-normal distribution of covariates causes difficulties with classical statistical modeling.

#### 3.1.1. Classification Model Database

**Figure 3:**
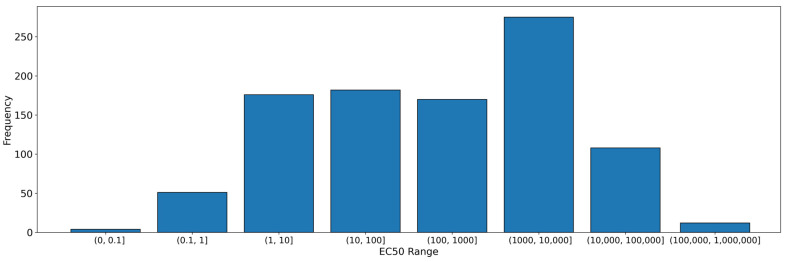
Distribution of EC_50_ values in different ranges in classification model.

The database of 978 chemical compound structures was constructed in the SMILES format and encoded information related to the assay and cell line. The symbols “≤”, “<” and “>” were removed from the EC_50_ values. For those molecules for which the EC_50_ was expressed as a range, the mean of the minimum and maximum values of the range was used in the dataset. The distribution of the final dataset for EC_50_ is shown in the figure below (Figure 3). The figure depicts the number of molecules within a given range. The database was divided into two classes, referred to as “high active” molecules and “low active” molecules. The first class, encoded as “1”, included molecules with EC_50_ values lower than or equal to 1000 nM, amounting to a total of 583 compounds. The second class, encoded as “0”, included molecules with EC_50_ values higher than 1000 nM, comprising a total of 395 compounds. The threshold was set at the stated level with the objective of identifying a potential drug with reduced biological activity.

#### 3.1.2. Regression Model Database

**Figure 4:**
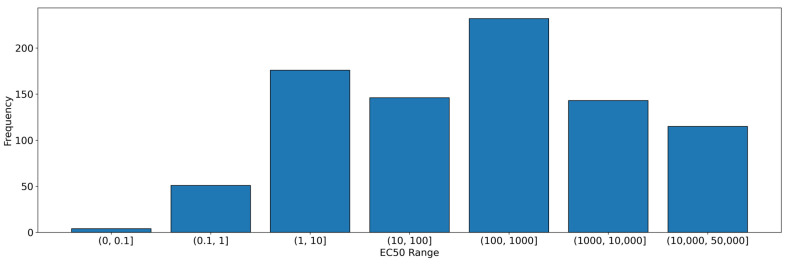
Distribution of EC_50_ values in different ranges in regression model.

In the case of the regression method, after numerous iterations, the most promising set was identified as follows: a training set of 701 molecules from the original database, where the EC_50_ values were signed with symbols “=”, “≤”, “<” and “>”, as described in Table 2. The test set comprised 166 molecules described as “=”. For all the aforementioned molecules with an EC_50_ value exceeding 50,000 nM, the EC_50_ value was estimated to be 50,000 nM. Molecules for which the EC_50_ value was expressed as a range of values were not included (Figure 4).

### 3.2. Molecular Descriptors

Mordred descriptors represent a comprehensive set of molecular descriptors utilized in the field of cheminformatics for the purpose of quantifying the structural and chemical properties of molecules. These descriptors are applicable to a wide range of molecular features, including constitutional, topological, geometrical and physicochemical properties [38]. They are frequently employed in the context of ML and QSAR modeling to forecast the behavior of molecules, such as their biological activity or chemical reactivity, based on their structural characteristics. The initial set of 2D-descriptors obtained through the use of Mordred packages comprised 1613 variables. In the event of missing data, the mean value of the respective column was used for substitution. Any columns that were empty or that exhibited a maximum value equal to the minimum value (i.e., a constant value) were removed. Ultimately, 1511 input variables related to 2D-descriptors were identified.

### 3.3. Obtained Models

#### 3.3.1. Classification Model

Modeling was performed with over 300 variations of the settings for the mljar tool. The optimal selected classification ensemble model was composed of eight smaller models, specifically 2 × Xgboost, 3 × RandomForest, LightGBM, CatBoost and ExtraTrees. The results of the classification model are illustrated in Table 3.

**Figure 5:**
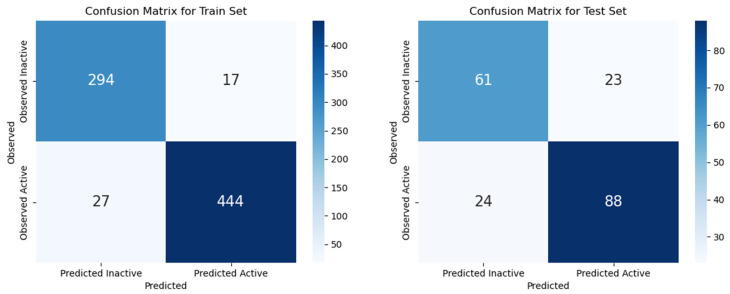
Confusion matrices for the training (**left**) and testing (**right**) sets, showing the classification performance for active and inactive molecules.

Moreover, experimental results were presented as confusion matrices and are shown in Figure 5. Based on the results, the model incorrectly predicted 44 molecules for the training set, representing 5.63% of the data. It is noteworthy that the majority of these (27 molecules) were active molecules that were incorrectly predicted as inactive. In contrast, for the test set, 24 and 23 molecules were incorrectly attributed to the observed class, representing a total of 24% of the test data.

#### 3.3.2. Regression Model

Modeling was performed with over 500 variations of the settings of the mljar tool. The regression model employed a subset of only 288 molecular descriptors, generated using the Mordred 2D-descriptor package, which provided a comprehensive representation of the molecular features. The resulting model performance is presented in Table 4. As a consequence of the unsatisfactory performance of the regression model, it was not subjected to further development.

### 3.4. SHAP Analysis

**Figure 6:**
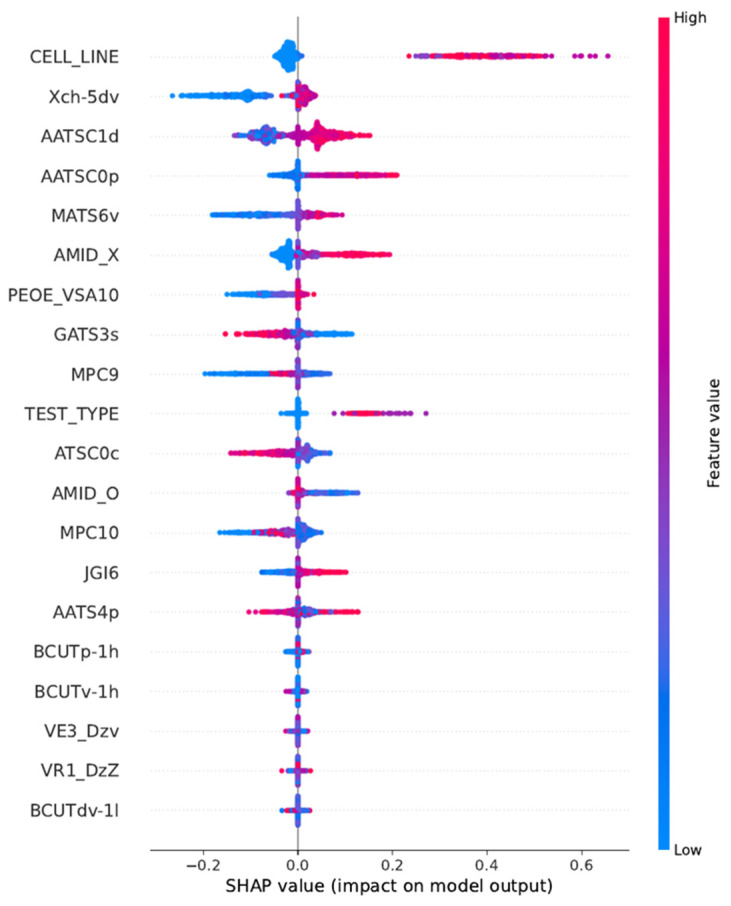
A summary of the SHAP analysis for the most important features. The color bar represents the range of the feature values: low (blue) and high (red).

According to the entire training dataset prepared for the classification model, Shapley’s analysis provided information on the relevant Mordred 2D-descriptors, as well as other features that were present in the database, i.e., information about the bioassays and cell lines utilized to obtain the EC_50_ values. The results are shown in Table 5, as well as in Figure 6 below.

A negative or positive SHAP value on the x-axis corresponds to a negative or positive effect of the relevant feature on the predicted output. The highest Shapley value was attributed to CELL_LINE, which suggests that the type of cell line, other than HepG2 which was encoded as “0”, used to obtain the EC_50_ result has a considerable influence on the model’s prediction. The contribution of TEST_TYPE, as indicated by Shapley’s value, is comparatively minor in relation to the more substantial factors, such as the cell line employed in the bioassay. In the classification model, low values of GATS3s, MPC9, ATSC0c and AMID_O are likely to increase output value. In contrast, the low values of Xch-5dv, AATSC1d, AATSC0p, MATS6v, AMID_X and PEOE_VSA10 tend to decrease results. For the applicability domain, ten Mordred 2D-descriptors were chosen: Xch-5dv, AATSC1d, AATSC0p, MATS6v, AMID_X, PEOE_VSA10, GATS3s, MPC9, ATSC0c and AMID_O. If the tested molecule is in the range of these descriptors based on the training set, the obtained prediction is reliable. The final result is dependent on a combination of factors, including chemical structure and physicochemical properties. The SHAP values assist in determining the influence of each factor on the predicted activity value.

### 3.5. Implementation

The classification model is provided in an online web application called “Aryl hydrocarbon receptor”, which is available at the link https://arylhydrocarbon-receptor.streamlit.app/, last accessed on 30 September 2024. The application has two modes, the first for single prediction and the second for batch calculations. In both cases, the user needs to provide a molecule’s SMILES. If researchers want to compare experimental results with our QSAR model, they should provide information about the assay type and cell line. The application will provider AhR activity class predictions, as well as information about the applicability domain. In the case of using confidential data, we recommend employing the local version available on the GitHub platform (https://github.com/nczub/AhR_Streamlit, last accessed on 30 September 2024) to run the application offline and in batch mode which is associated with a shorter analysis time.

## 4. Discussion

This work demonstrates once more that the diversity of AhR modulators represents a significant barrier to the development of a valuable QSAR model. The assembly of a comprehensive and diverse database comprising AhR ligands required considerable time, due to limited and fragmented information available in existing databases and the existing literature. Patents were also instrumental sources for the construction of the database. A considerable drawback was the wide range of EC_50_ values, which had a detrimental impact on the model, hampering further development. Moreover, a substantial proportion of the database reported only range values, which further increased the difficulty of developing a high-performance model. The use of molecules for which the EC_50_ value has not been quantified but only approximated frequently entailed making assumptions that could often significantly overestimate the molecule’s EC_50_ value, thereby impeding the development of a robust regression model with optimal performance.

In order to identify the model that fits database specifications the best, available classification and regression techniques were found to be most appropriate. The selected classification model used the entire database, with a cutoff threshold of 1000 nM. Due to the uneven distribution between the classes, the classification database was unbalanced. Specifically, the records in class “1” represent 60% of the database. The model demonstrated satisfactory performance in terms of accuracy and the capacity to differentiate between classes, by achieving an overall accuracy of 0.760, precision of 0.793, recall of 0.786 and F1 score of 0.789.

On the other hand, the effort to develop a regression model with the potential to markedly enhance the search for new AhR receptor ligands by predicting the EC_50_ value led to unsatisfactory outcomes. In fact, the existing information regarding AhR ligands was insufficient to develop an effective database. Attempts were made to modify the previously prepared database. For the regression model, lower NRMSE values are preferable. A value of 10.89% indicates that the model has moderate accuracy and the error is relatively small, yet there is still considerable space for improvement. The coefficient of determination R² of 0.208 shows that the model explains only approximately the 20% of the observed variation. This value is relatively low, suggesting that the model has difficulty in capturing the full range of patterns in the data. The model’s inability to accurately represent the data raises concerns about its continued use and the reliability of the results obtained.

The SHAP study, conducted on the training set for the classification model, revealed information on Mordred descriptors and database features. Noticeably, cell line and assay types were among the selected features used to determine EC_50_ values, indicating that these are critical parameters affecting model performance.

The integration of ML models into the drug discovery process offers a multitude of advantages that can markedly enhance and expedite the entire process of identifying novel active compounds. The construction of a web-based application designated as Aryl Hydrocarbon Receptor represents an initial stage in a novel approach to researching new activators of the AhR.

## 5. Conclusions

In an attempt to develop a predictive model that can estimate AhR modulation, a database comprising 978 molecules and their respective EC_50_ values was constructed. Classification and regression models were built. The former’s best accuracy and F1 scores were 0.760 and 0.789, respectively, and it sorted molecules based on a predicted EC_50_ cutoff of 1000 nM. The latter provided an RMSE of 5444 and an R^2^ of 0.208 for the test set. The SHAP analysis on the training set prepared for the classification model identified the Mordred 2D-descriptors most relevant to the prediction result and revealed the positive impact of the cell line type utilized in the bioassay to determine the EC_50_ value. The classification model was implemented into a publicly available online application.

While far from optimal, these results may be helpful for triggering further research into the development of a QSAR tool enabling further comprehension of the implications of ligand structure on AhR activity modulation.

## Figures and Tables

**Table 1:** Molecules by cell line and test type.

	HepG2	HT29	Huh-7	U937	MCF7	HEK293
Luminescence assay	629	24	51	98	0	0
Fluorescence EROD assay	19	0	0	0	9	0
Fluorescence NT assay	0	0	0	0	0	148

**Table 2:** Number of molecules with description of EC_50_ value (nM).

EC_50_ Value (nM)	Molecule Number
“=” (numerical format)	415
“<0.014”	1
“≤10”	95
“≤1000”	36
“>100”	71
“>370”	8
“>1000”	129
“>3300”	3
“>6700”	1
“>10,000”	20
“>30,000”	59
“>50,000”	2
“>91,000”	6
“>100,000”	9
“>200,000”	3
“>400,000”	1
“>1,000,000”	8
“10 < EC_50_ ≤ 100”	36
“100 < EC_50_ ≤ 1000”	62
“1000 < EC_50_ ≤ 10,000”	8
“10,000 < EC_50_ ≤ 100,000”	5

**Table 3:** Results of classification for train and test sets.

Dataset	Accuracy	Precision	Recall	F1	MCC
Train set	0.944	0.963	0.943	0.953	0.883
Test set	0.760	0.793	0.786	0.789	0.511

**Table 4:** Train and test set evaluation metrics for regression model.

Dataset	RMSE	NRMSE	R^2^
Train set	7328	14.66%	0.673
Test set	5444	10.89%	0.208

**Table 5:** Shapley values for the most important features.

Feature	Shapley Value	Description
CELL_LINE	0.13564	Type of human cell line used to obtain EC_50_ value
Xch-5dv	0.06370	Five-ordered Chi chain weighted by valence electrons
AATSC1d	0.05708	Averaged and centered Moreau–Broto autocorrelation of lag 1 weighted by sigma electrons
AATSC0p	0.04938	Averaged and centered Moreau–Broto autocorrelation of lag 0 weighted by polarizability
MATS6v	0.04231	Moran coefficient of lag 6 weighted by vdw volume
AMID_X	0.03896	Averaged molecular ID on halogen atoms
PEOE_VSA10	0.03757	MOE Charge VSA Descriptor 10 (0.10 ≤ x < 0.15)
GATS3s	0.03390	Geary coefficient of lag 3 weighted by intrinsic state
MPC9	0.02870	Nine-ordered path count
TEST_TYPE	0.02831	Type of bioassay used to obtain EC_50_ value
ATSC0c	0.02759	Centered Moreau–Broto autocorrelation of lag 0 weighted by gasteiger charge
AMID_O	0.02707	Averaged molecular ID on O atoms
MPC10	0.02685	Ten-ordered path count
JGI6	0.02017	Six-ordered mean topological charge
AATS4p	0.01801	Averaged Moreau–Broto autocorrelation of lag 4 weighted by polarizability
BCUTp-1h	0.00281	First heighest eigenvalue of Burden matrix weighted by polarizability
BCUTv-1h	0.00183	First heighest eigenvalue of Burden matrix weighted by vdw volume
VE3_Dzv	0.00140	Logarithmic coefficient sum of last Eigenvector from Barysz matrix weighted by vdw volume
VR1_DzZ	0.00138	Randic-like eigenvector-based index from Barysz matrix weighted by atomic number
BCUTdv-1l	0.00137	First lowest eigenvalue of Burden matrix weighted by valence electrons

## Data Availability

The data presented in this study are available on request from the corresponding author.

## Supplementary Materials

The following supporting information can be downloaded at: https://www.mdpi.com/article/10.3390/pharmaceutics16111456/s1, Table S1: The database of 978 molecules used in this study.
